# Combination of interferon-alpha and 5-fluorouracil inhibits endothelial cell growth directly and by regulation of angiogenic factors released by tumor cells

**DOI:** 10.1186/1471-2407-9-361

**Published:** 2009-10-12

**Authors:** Hiroshi Wada, Hiroaki Nagano, Hirofumi Yamamoto, Takehiro Noda, Masahiro Murakami, Shogo Kobayashi, Shigeru Marubashi, Hidetoshi Eguchi, Yutaka Takeda, Masahiro Tanemura, Koji Umeshita, Yuichiro Doki, Masaki Mori

**Affiliations:** 1 Department of Surgery, Graduate School of Medicine, Osaka University, 2-2 Yamadaoka E-2, Suita 565-0871 Osaka, Japan

## Abstract

**Background:**

The combination therapy of interferon (IFN)-alpha and 5-fluorouracil (5-FU) improved the prognosis of the patients with hepatocellular carcinoma (HCC). To determine the molecular mechanisms of the anti-tumor and anti-angiogenic effects, we examined the direct anti-proliferative effects on human umbilical vein endothelial cells (HUVEC) and indirect effects by regulating secretion of angiogenic factors from HCC cells.

**Methods:**

The direct effects on HUVEC were examined by TUNEL, Annexin-V assays and cell cycles analysis. For analysis of the indirect effects, the apoptosis induced by the conditioned medium from HCC cell treated by IFN-alpha/5-FU and expression of angiogenic factors was examined.

**Results:**

IFN-alpha and 5-FU alone had anti-proliferative properties on HUVEC and their combination significantly inhibited the growth (compared with control, 5-FU or IFN alone). TUNEL and Annexin-V assays showed no apoptosis. Cell cycle analysis revealed that IFN-alpha and 5-FU delayed cell cycle progression in HUVEC with S-phase accumulation. The conditioned medium from HuH-7 cells after treatment with IFN/5-FU significantly inhibited HUVEC growth and induced apoptosis, and contained high levels of angiopoietin (Ang)-1 and low levels of vascular endothelial growth factor (VEGF) and Ang-2. Knockdown of Ang-1 in HuH-7 cells abrogated the anti-proliferative effects on HUVEC while knockdown of Ang-2 partially rescue the cells.

**Conclusion:**

These results suggested that IFN-alpha and 5-FU had direct growth inhibitory effects on endothelial cells, as well as anti-angiogenic effects through regulation of angiogenic factors released from HCC cells. Modulation of VEGF and Angs secretion by IFN-alpha and 5-FU may contribute to their anti-angiogenic and anti-tumor effects on HCC.

## Background

Hepatocellular carcinoma (HCC) is one of the most common malignancies worldwide, especially in Eastern Asia. Advancements in diagnostic biotechnology and new therapeutic modalities have improved the prognosis of patients with small HCC. However, the entire prognosis of patients with HCC is still poor, particularly in patients with tumor thrombi in the major trunk of the portal vein, because HCC can invade the portal vein in the early period and cause intrahepatic metastases. Although chemotherapy commonly plays a central role in the treatment of advanced stage HCC, no standard treatment regimen has been established yet [1], because of resistance of such tumors to anti-cancer drugs [2]. Recently, we and others reported that the combination of interferon (IFN) and chemotherapeutic agents for advanced HCC resulted in excellent clinical outcome [3-6]. The clinical response rate (CR and PR ration) of patients with unresectable advanced HCC and portal vein tumor thrombosis to the combination therapy of IFN-α and hepatic arterial infusion of 5-fluorouracil (5-FU) is about 50% [6]. Furthermore, combining this therapy with surgery can reduce recurrence [3,4].

The exact mechanism of action of this combination therapy is not clear at present. The IFNs are a family of natural glycoproteins and regulatory cytokines with pleiotropic cellular functions, such as anti-viral, anti-proliferative and immunomodulatory activities [7-9]. IFN-α enhances the anti-tumor effects of 5-FU by regulating thymidine phosphorylase and accumulation of fluorodeoxyuridine monophosphate (FdUMP) caused by inhibition of thymidylate [10]. We reported previously that the expression of IFN-α/β receptor correlates with the growth-inhibitory activity and that IFN-α and 5-FU synergistically inhibit cell proliferation, induced cell cycle arrest [11,12], and induce apoptosis by regulating the expression of apoptosis-related molecules [13]. IFN-α also has immunomodulatory properties and the tumor necrosis factor-related apoptosis inducing ligand (TRAIL) or Fas/Fas-L pathway partially contributes to the anti-tumor effects of IFN-α/5-FU combination therapy [14,15]. On the other hand, IFNs also has significant antitumor activity through the inhibition of angiogenesis in experimentally-induced tumors in animals [16]. Specifically, IFNs regulates the transcription and production of pro-angiogenic molecules, such as vascular endothelial growth factor (VEGF) [17,18], basic fibroblast growth factor (b-FGF) [19], matrix metalloproteinase (MMP)-2 and MMP-9 [20,21], and interleukin (IL)-8 [22]. Marschall *et al*. [17] recently reported that the therapeutic effects of IFN-α on neuroendocrine tumor cells were based on Sp1- and/or Sp3-mediated inhibition of VEGF transcription both *in vivo *and *in vitro*. We also reported recently that IFN-α and 5-FU combination therapy synergistically inhibited tumor angiogenesis *in vivo *and their effects correlated with regulation of VEGF and angiopoietins (Angs) [16].

The present study is an extension to the above studies and was designed to determine the direct effects of IFN-α and 5-FU on endothelial cells, using cultured human umbilical vein endothelial cells (HUVEC). Moreover, we also determined the indirect effects of IFN-α and 5-FU on endothelial cells mediated through various angiogenic factors secreted by HCC cell lines and examined their effects on endothelial cells, with a special focus on VEGF and Angs.

## Methods

### Cell lines and reagents

HCC cell line HuH7 was maintained as an adherent monolayer in Dulbecco's modified Eagle's medium (DMEM) supplemented with 10% fetal bovine serum (FBS) and 1% penicillin-streptomycin mixture. HUVEC were grown on MCDB131 culture medium (Chlorella, Inc., Tokyo, Japan) supplemented with 5% fetal bovine serum, antibiotics and 10 ng/mL basic fibroblast growth factor (bFGF). Cell cultures were grown on plastic plates and incubated at 37°C in a mixture of 5% CO_2 _and 95% air. Purified human IFN-α was obtained from Otsuka Pharmaceutical Co. (Tokushima, Japan) and purified 5-FU was obtained from Kyowa Hokko Co. (Tokyo).

### Growth inhibitory assay

HUVEC (1 × 10^4 ^cells per well) were added in triplicate to a 96-well microplate, and after overnight incubation, the medium was replaced with 0.1 ml of fresh medium containing various concentrations of 5-FU and/or IFN-α. HUVEC cells suspended in complete medium were used as control for cell viability. After 72-hour treatment, the number of viable cells was assessed by the 3-(4-, 5-dimethylthiazol-2-yl)-2, 5-dyphenyl tetrazolium bromide (MTT) (Sigma Co, St. Louis, MO) assay as reported previously [12]. Briefly, 10 μl (50 μg) of MTT were added to each well. The plate was incubated for 4 h at 37°C, followed by removal of the medium and addition of 0.1 ml of 2-propanol to each well to dissolve the resultant formazan crystals. Plate absorbance was measured in a microplate reader at a wavelength of 570 nm. These assays were repeated three times, and similar results were obtained. In other parts of the present study, experiments were repeated at least twice, and no discrepant results were obtained.

Growth curves for each treatment were constructed as follow. Cells were uniformly seeded in triplicates into 6-well dishes. Twenty-four hours later (day 0), the culture medium was replaced with 3 ml of fresh medium with or without 5-FU (0.5 μg/ml) and IFN-α (500 units/ml). The medium was changed every 48 h, and on days 1, 3, 5 and 7, cell numbers were counted using a hemocytometer by trypan blue dye exclusion.

### Cell cycle analysis

Flow cytometric analysis was performed as described previously [12]. Briefly, cells were washed twice with PBS and fixed overnight in 70% ethanol before being washed and resuspended in 1 ml of PBS. Propidium iodide (Sigma-Aldrich, St. Louis, MO) and RNase (Nippon Gene, Tokyo) were added for 30 min at 37°C. Samples were filtered through 44 μm nylon mesh and data were acquired with a FACSort (Becton Dickinson Immunocytometry Systems, San Jose, CA). Analysis of the cell cycle was carried out using ModFit software (Becton Dickinson).

### BrdU labeling index

Cells were incubated with 20 μmol/L BrdUrd (Sigma-Aldrich) at 37°C for 30 minutes and fixed in 70% cold ethanol for 30 minutes. After quenching endogenous peroxidase activity, the slides were incubated in 4 N HCl at 37°C for 5 minutes and neutralized with buffered boric acid (pH 9.0) for 5 minutes. After blocking with 10% rabbit serum, anti-BrdUrd antibody (DAKO, Glostrup, Denmark) was applied to the slides at 1:20 dilution at room temperature for 2 hours followed by the avidin-biotin complex method. For quantification, five microscopic fields were randomly selected at high power magnification (× 200) and the percentage of BrdU-positive cells was calculated as described previously [23].

### Detection of apoptosis

To detect apoptosis, we used the terminal deoxynucleotidyl transferase-mediated dUTP nick end-labeling (TUNEL) method using the Apop Tag *in situ *apoptosis detection Kit (Chemicon International, Inc., Temecula, CA) as described previously [13]. This method can detect fragmented DNA ends of apoptotic cells. Briefly, the paraffin-embedded sections were deparaffinized in xylene and rehydrated in a graded series of ethanol baths. The sections were treated with 20 μg/ml of proteinase K in distilled water for 10 min at room temperature. The adherent cultured HUVEC cells were fixed in 1% paraformaldehyde for 10 minuets. To block endogenous peroxidase, the slides incubated in methanol containing 0.3% hydrogen peroxide for 20 min. The remaining procedures were performed according to the instructions provided by the manufacturer. For quantification of apoptosis, five microscopic fields were randomly selected at high power magnification (× 200) and the average counts of TUNEL-positive cells were calculated.

The binding of annexin V-FITC was also used as a sensitive method for measuring apoptosis, according to the method described previously [14]. Briefly, after treatment with IFN-α and/or 5-FU, the cultured cells (1 × 10^6^) were incubated with binding buffer (10 mM HEPES, 140 mM NaCl and 2.5 mM CaCl_2_, pH 7.4) containing saturating concentrations of annexin V-FITC (BioVision Research Products, Mountain View, CA) and propidium iodide (PI) for 15 min at room temperature. After incubation, the cells were pelleted and analyzed on a FACScan (BD), and data were processed using Cell Quest™ software (BD).

### *In vitro *angiogenesis assay

*In vitro *formation of tubular structures in HUVEC was examined using *in vitro *Angiogenesis Assay kit (Chemicon). HUVEC cells were seeded on Matrigel-coated well and maintained on complete medium. After attachment of the cells on Matrigel, the medium was changed with fresh medium, either with or without the recombinant VEGF protein (25 ng/mL), and incubated for 12 hours. Cells were then observed under an inverted microscope and the number of capillary structures was counted as reported previously [23] and according to recommended procedure by the respective manufacturer, we counted the capillary tube branch points in ten random view-fields per well and calculated the average of branch points.

### Effect of conditioned medium from cancer supernatants on HUVEC proliferation

To evaluate anti-angiogenic effects mediated by angiogenic factors released from cancer cells, we used supernatants from HuH-7 in subsequent experiments. To obtain supernatants from cultured cancer cells as conditioned medium (CM), HuH-7 cells were seeded on 150-mm dishes containing medium with 10% FBS. After 24 hours, the medium was replaced with serum-free UltraCulture medium (Calbiochem, La Jolla, CA), containing IFN-α (500 IU/ml) and/or 5-FU (0.5 μg/ml). The medium was collected after 48 hours. Then, HUVEC were cultured in CM in each treatment and their proliferation was evaluated by MTT assay and the frequency of apoptosis by TUNEL assay.

### ELISA assays for VEGF, Ang-1 and Ang-2 in cell culture supernatants

HuH7 cells (3 × 10^4^) were seeded into 12-well plates and incubated overnight. After overnight incubation, the culture medium was removed and replaced with 2 ml of DMEM with or without 5-FU (0.5 μg/ml) and IFN-α (500 IU/ml). The conditioned medium in each group was collected after 48 h. VEGF, Ang-1 and Ang-2 levels were analyzed using the human VEGF enzyme-linked immunosorbent assay (ELISA) kit (Biosource International, Camarillo, CA), the Quantikine human Ang-1 ELISA kit (R&D Systems, Minneapolis, MN) and the Quantikine human Ang-2 ELISA kit (R&D Systems), respectively. These ELISA assays were performed as recommended by the respective manufacturer.

### Ang-1 or Ang-2 specific siRNA knockdown

SiTrio Ang-1, Ang-2 and negative control small interfering RNA (siRNA) were purchased from B-Bridge International, Inc. (Sunnyvale, CA). Each siRNA consisted of three different target sequences; which were as follows: negative control 5 TCCGCGCGATAGTACGTA- 3, 5-TTACGCGTAGCGTAATACG-3, and 5 ATTCGCGCGTATAGCGGT-3 and siRNA Ang-1 human, 5-CGCUGGAGCCCGUGAAAAATT- 3, 5-CCAGAGUGAUCAAGUGUGATT- 3, and 5-UCCAAUAGGUGUAGGAAAUTT-3. Cells were transfected with 100 nmol/L siRNA using LipofectAMINE 2000 (Invitrogen, Carlsbad, CA) in Opti-MEM I Reduced Serum Medium (Invitrogen). After 6 hours, medium was replaced by standard medium. At 24 hours after transfection, the medium was replaced with serum-free medium with or without IFN-α (500 IU/ml) and/or 5-FU (0.5 μg/ml). These media were collected after 48 hours, and HUVEC were cultured in each medium followed by evaluation of proliferation by MTT assay and frequency of apoptosis by TUNEL assay.

### Statistical analysis

Data are expressed as mean ± SD. Statistical analysis was performed using the StatView J-4.5 program (Abacus Concepts, Inc., Berkeley, CA). The unpaired Student's *t*-test was used to examine differences in cell proliferation, apoptosis, BrdUrd labeling index and the expression of VEGF, Ang-1, Ang-2 proteins between each group. A *p *level less than 0.05 was considered statistically significant.

## Results

### Anti-proliferative effects of IFN/5-FU on HUVEC

**Figure 1:**
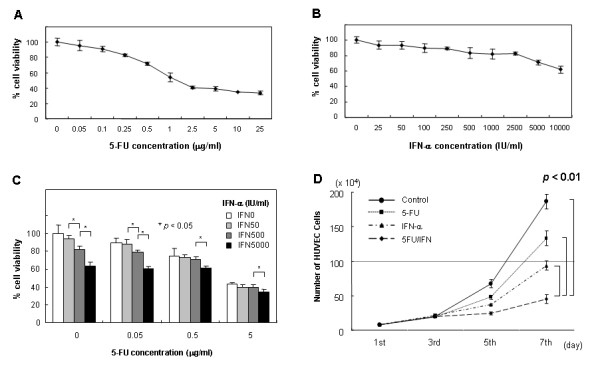
**MTT growth inhibitory assay**. 5-FU alone inhibited HUVEC cells growth (A). IFN-α alone slightly inhibited HUVEC cell growth, even when used at a high concentration (10,000 units/ml) (B). Significant synergistic effects for IFN-α and 5-FU were observed at 0.05 μg/ml of 5-FU and 500 or 5,000 units/ml of IFN-α (p < 0.05), but not at 0.5 or 5 μg/ml of 5-FU plus 500 units/ml of IFN-α (C). A significant difference was observed in cell numbers on day 7 between the IFN/5-FU combination group and the other groups (D).

To evaluate the effect of IFN-α and 5-FU on proliferation of HUVEC, we performed growth inhibitory assays by MTT assay. Cells were exposed to 5-FU and/or IFN-α for 72 hours at various concentrations. 5-FU alone inhibited HUVEC cells growth (Figure 1A); the IC_50 _of 5-FU was 3.06 ± 0.48 μg/ml. IFN-α alone slightly inhibited HUVEC growth, but even at high concentrations (10,000 units/ml), IFN-α moderately reduced cell growth to 62.0 ± 4.5% (Figure 1B). When IFN-α and 5-FU were used simultaneously at various concentrations, significant effects were observed at 0.05 μg/ml of 5-FU plus 500 or 5,000 units/ml of IFN-α (p < 0.05). However, these effects were not observed with 0.5 or 5 μg/ml of 5-FU plus 500 units/ml of IFN-α (Figure 1C). Growth curves were constructed up to 7 days (Figure 1D). The doubling times were 29.7, 34.2, 45.5 and 78.9 h for cultures of control, 5-FU alone, IFN-α alone and 5-FU plus IFN-α, respectively. A significant difference was observed in cell numbers at day 7 between the IFN/5-FU combination group and other groups.

### Cell cycle analysis

**Figure 2:**
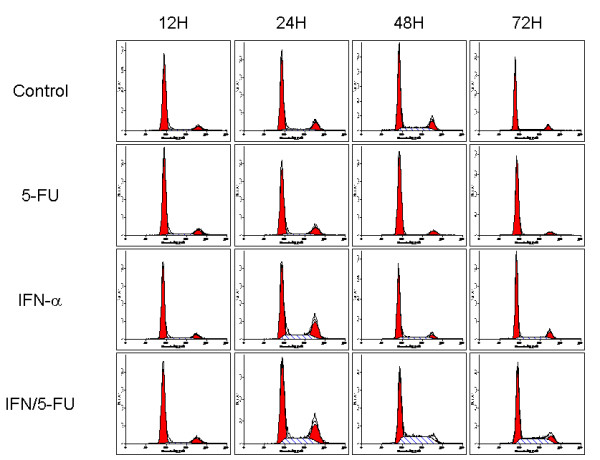
**Flow cytometric analysis of cell cycle progression in HUVEC cells treated with or without IFN-α (500 units/ml) and/or 5-FU (0.5 μg/ml)**. To synchronize the cell cycle in G0-G1, HUVEC cells were first pre-treated with 2 μM aphidicolin for 16 h. Cells were collected 12, 24, 48 and 72 h later. After pre-treatment by aphidicolin, the majority of cells (86.3%) were in G0-G1. At 24 h, IFN-α alone and IFN/5-FU increased the number of cells with S-phase DNA content. At 48 h and 72 h, IFN/5-FU still resulted in S-phase accumulation.

Next, we performed flow cytometric analyses to examine changes in cell cycle progression when HUVEC cells were treated with or without IFN-α (500 units/ml) and/or 5-FU (0.5 μg/ml). To synchronize the cell cycle in G0-G1, HUVEC cells were pre-treated by 2 μM aphidicolin (Sigma-Aldrich) for 16 h before the addition of IFN-α/5-FU. Cells were then collected 12, 24, 48 and 72 h later. Flow cytometric data confirmed that after pre-treatment with aphidicolin, the majority of cells (86.3%) were in G0-G1. At 24 h, IFN-α alone and IFN/5-FU increased the number of cells with S-phase DNA content. At 48 and 72 h, IFN/5-FU still showed S-phase accumulation (Figure 2). These results suggest that IFN-α can regulate the cell cycle and that IFN/5-FU delayed the cell cycle of HUVEC in the S-phase.

### BrdUrd labeling index

**Figure 3:**
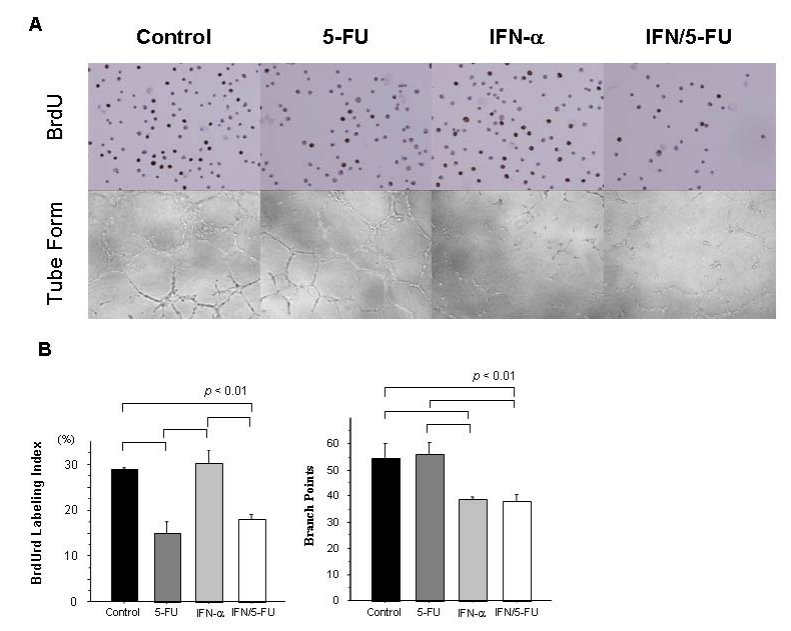
**BrdUrd labeling index and tube formation *in vitro***. (A) *In vitro *angiogenesis assay showed that HUVEC formed vessel-like structures (tubes) when plated on Matrigel-coated wells. 5-FU treatment did not inhibit tube and network formation. In contrast, IFN-α caused thinner or only weakly-stained tube-like structures. IFN/5-FU also inhibited tube formation compared to the control and caused only weak staining of the tube-like structures, similar to IFN-α alone. (B) 5-FU alone and IFN/5-FU caused significant decreases in BrdUrd labeling index compared with the control and IFN-α alone (*p *< 0.01). There was no difference in the index between 5-FU alone and IFN/5-FU combination (A, B). There was a significant difference in the number of capillary connections, defined as cross-points consisting of three tubes, among the control, 5-FU alone and IFN-α, IFN/5-FU (*p *< 0.01).

We also assessed cell growth and DNA synthesis using BrdUrd. 5-FU alone and IFN/5-FU caused a significant decrease in BrdUrd labeling index than control and IFN-α alone (*p *< 0.01). There was no difference in BrdUrd labeling index between 5-FU alone and the combination of IFN/5-FU (Figure 3A, B). These results suggest that 5-FU inhibits DNA synthesis in HUVEC.

### IFN-α directly inhibits tube formation *in vitro*

*In vitro *angiogenesis assay showed that HUVEC formed vessel-like structures (tubes) when plated on Matrigel-coated wells (Figure 3A). 5-FU treatment did not inhibit tube formation or network formation. In contrast, thin or only weakly-stained tube-like structures were noted in the presence of IFN-α. The combination of IFN/5-FU also inhibited tube formation compared to the control and their presence was associated with only weakly-stained tube-like structures, similar to IFN-α alone. There was a significant difference in the number of capillary connections, defined as cross-points consisting of three tubes among the control, 5-FU alone and IFN-α, IFN/5-FU (*p *< 0.01; Figure 3B). These results suggest that IFN-α suppresses HUVEC tube formation and that 5-FU does not cause further inhibition of this action.

### IFN/5-FU do not directly induce HUVEC apoptosis

**Figure 4:**
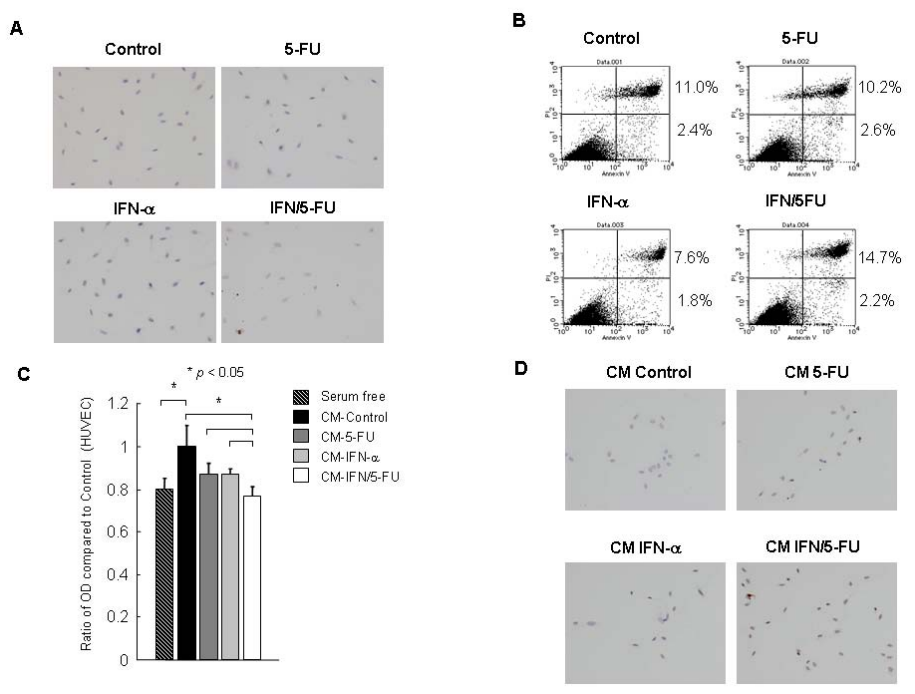
**Effect of 5-FU alone and IFN-α, IFN/5-FU on apoptosis of HUVEC**. (A) TUNEL assay showed limited number of TUNEL-positive cells in each treatment. (B) IFN/5-FU did not induce apoptosis of HUVEC in annexin-V assay. The percentage of Annexin-V positive cells is shown in figures. (C) Serum-free CM from control culture of HuH-7 significantly promoted HUVEC growth. Supernatants from HuH-7 treated by IFN-α (500 units/ml) and 5-FU (0.5 μg/ml) (CM-IFN/5-FU) significantly inhibited the growth of HUVEC. (D) Growth inhibition of CM-IFN/5-FU was due to induction of apoptosis. The number of TUNEL-positive cells in CM-IFN/5-FU was significantly higher than in other conditioned media (Figure 4D).

To examine whether the anti-proliferative effects of IFN/5-FU on HUVEC represent induction of apoptosis, we used TUNEL assay and Annexin V assay. TUNEL assay showed that TUNEL-positive cells were hardly found in each treatment at all (Figure 4A). To confirm these results, we performed the annexin-V assay to detect pre-apoptotic cells. Similarly, IFN/5-FU did not induce HUVEC apoptosis (Figure 4B).

### CM from HuH-7 treated by IFN/5-FU inhibits HUVEC growth

Next, we investigated the anti-angiogenic effects of angiogenic factors secreted by cancer cells using supernatants from HuH-7 as the conditioned medium (CM). Compared to serum-free medium, CM from control cultures of HuH-7 cells significantly promoted HUVEC growth. The supernatants of IFN-α (500 units/ml) and 5-FU (0.5 μg/ml) treated HuH-7 cells (CM-IFN/5-FU) significantly inhibited the growth of HUVEC (Figure 4C). There were significant differences between CM-IFN/5-FU and each of CM-control, CM-IFN and CM-5-FU. In the next step, we confirmed by TUNEL assay, that the growth inhibition of CM-IFN/5-FU was related to induction of apoptosis (Figure 4D). The number of TUNEL-positive cells in CM-IFN/5-FU was significantly higher than that in the other conditioned media.

### IFN-α/5-FU inhibit VEGF and Ang-2 and enhance Ang-1 production *in vitro*

**Figure 5:**
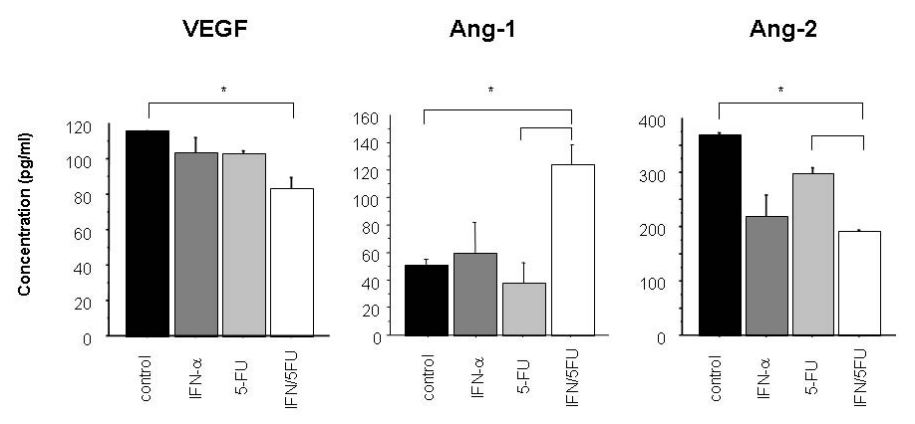
**Concentrations of angiogenic factors (VEGF, Ang-1 and Ang-2) in the supernatants of HCC cells treated without (control) or with IFN-α, 5-FU or their combination, measured by ELISA assay kits**.

We also examined the angiogenic factors (VEGF, Ang-1 and Ang-2) secreted in the supernatant of HCC cells using the respective ELISA kits. Treatment of cells with the combination of IFN-α and 5-FU resulted in a significant reduction in the concentration of secreted VEGF and Ang-2 and increased secretion of Ang-1 in culture supernatant compared with the control (Figure 5).

### Ang-1 or Ang-2 knockdown abrogates anti-proliferative effects of conditioned medium

**Figure 6:**
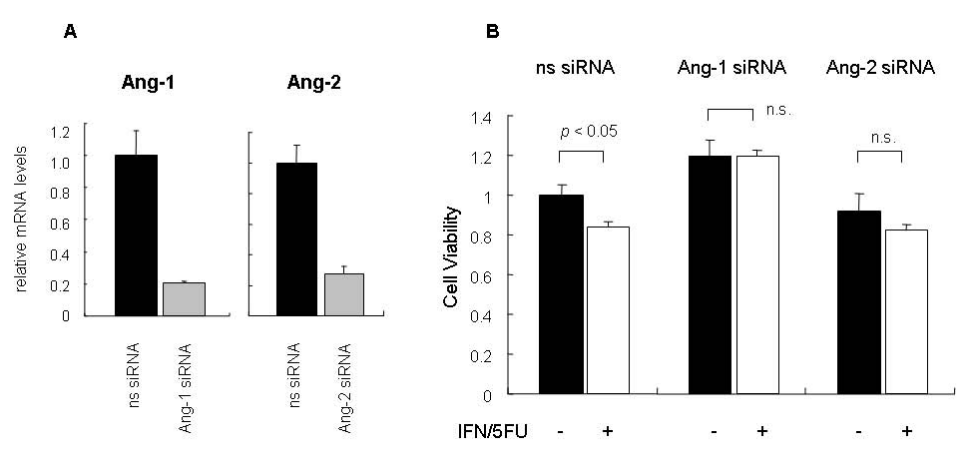
**(A) Knock-down of Ang-1 or Ang-2 efficiently represses the expression of Ang-1 or Ang-2 mRNA in HuH-7 cells**. HuH-7 cells were transfected to Ang-1 or Ang-2 siRNA. Forty eight hours after the transfection, we evaluated the expression of Ang-1 or Ang-2 mRNA by real time RT-PCR. Values shown are relative induction of the indicated genes. (B) The supernatant of HuH-7 cells after knockdown of Ang-1 completely abrogated the anti-proliferative effects of the conditioned medium from IFN/5-FU treated HuH-7 cells. HuH-7 cells treated with siRNA for Ang-1, Ang-2 or non-specific for 24 hours, and then we collected the supernatant after treatment with or without IFN/5-FU for 48 hours. We evaluated viability of HUVEC cells by MTT assay. The percentage of viable cells was significantly reduced by the conditioned media after the treatment of IFN/5-FU. There is no significant difference between the conditioned media after treatment with or without IFN/5-FU after knockdown of Ang-1 or Ang-2.

To determine that angiopoietins from IFN/5-FU-treated tumor cells mediated the observed proliferative and apoptotic effects, we evaluated whether endogenous expression of Ang-1 and Ang-2 are required anti-proliferative effects of the supernatants of IFN/5-FU treated HuH-7 cells using Ang-1 and Ang-2 siRNAs. Transfection of Ang-1 or Ang-2 siRNA into HuH-7 cells clearly down-regulated their expression to less than 30% of the control (Figure 6A). Knockdown of Ang-1 completely abrogated the anti-proliferative effects of the CM from IFN/5-FU-treated HUVEC, while Ang-2 knockdown partially rescued HUVEC growth (Figure 6B). These results suggest that the combination of IFN and 5-FU regulates the expression of angiopoietins and Ang-1 and Ang-2 mediate, at least in part, the anti-angiogenic effects of this combination therapy for HCC.

## Discussion

In HCC, as part of the remodeling of the hepatic structures from normal liver to cirrhotic liver, inflammation and tissue reconstruction stimulates angiogenesis. Furthermore, HCC is known as one of the most hypervascular tumors and angiogenesis is necessary for its development. Solid tumors cannot grow beyond 2-3 mm without new blood vessels due to lack of oxygen and nutrients [24]. Once angiogenesis starts, the tumor grows rapidly, invades other organs and metastasizes to remote sites [25]. This is a multi-step process regulated by a balance between inducers and inhibitors of endothelial cells proliferation and migration. These pro- and anti- angiogenic molecules are produced by tumors and host components cells [26]. To date, many factors known to promote or inhibit angiogenesis have been identified, including growth factors, cytokines and proteases [27]. Previous studies showed that IFN-α has anti-angiogenic properties in various tumors such as Kaposi's sarcomas [28], infantile hemangiomas [29] and some vascular-rich malignancies, melanoma, renal cell carcinoma and neuroendocrine tumors [30]. Therefore, we focused in the present study on the anti-angiogenic effects of the combination of IFN-α and 5-FU to determine the mechanism of action.

Firstly, we examined whether IFN-α or 5-FU has anti-proliferative properties on endothelial cells using HUVEC. The results of MTT assay showed that 5-FU significantly inhibited HUVEC growth; while IFN-α had mild short-term growth inhibitory effects even when used at a high dose. To evaluate the long-term effects of IFN-α or the synergistic anti-proliferative effects of IFN-α and 5-FU on endothelial cells, we performed cell growth assay by cell counts methods. At 500 IU/ml, IFN-α alone significantly inhibited HUVEC growth on 7th day, compared to the control.

Furthermore, the combination of IFN-α and 5-FU significantly inhibited the growth of HUVEC on 5th and 7th day, compared to the control, 5-FU or IFN-α alone. IFNs have multiple biological actions causing modulation of gene expression, immunomodulation and regulation of cell cycle. IFNs also inhibit endothelial cell growth *in vitro*. Our results are consistent with those of previous reports, which showed that IFNs has anti-proliferative properties on endothelial cells [31,32]. Moreover, the combination of IFN-α and 5-FU synergistically inhibited HUVEC growth. In this regard, Solorzano *et al*. [18] reported that the combination with IFN and gemcitabine synergistically induced endothelial cell apoptosis using *in vivo *orthotopical pancreas cancer models.

Is the growth inhibitory effect of IFN/5-FU due to induction of apoptosis, cell cycle arrest or inhibition of DNA synthesis? To answer this question, we used the BrdUrd labeling assay and showed that 5-FU significantly inhibited DNA synthesis 24 hours after the administration; although there was no apparent difference in the BrdUrd labeling index between control and IFN-α alone. TUNEL assay and Annexin-V assay showed that none of the agents used induced apoptosis of endothelial cells. These results are in agreement with those of Hong et al [31] who reported that IFN-α significantly inhibited HUVEC growth but did not induce apoptosis of IFN-α treated HUVC. We also reported previously that IFN/5-FU did not induce apoptosis of cultured normal liver epithelial cells *in vitro *or hepatocytes in patients who underwent hepatectomy after IFN/5-FU combination therapy, although the combination treatment induced apoptosis of tumor cells [14] These results suggest that non-cancerous cells are resistant to apoptosis induced by IFN/5-FU. Analysis of cell cycle progression in HUVEC provided a clue to the anti-proliferative mechanism of IFN/5-FU on endothelial cells. A marked delay in cell cycle progression was found in IFN/5-FU-treated cells with S-phase accumulation. In cells treated with IFN-α only, a slight accumulation of cells at the S-phase was also detected. The link between cell cycle regulation and IFN has been reported previously. IFN-α is reported to induce G1 phase arrest in murine fibroblasts (NIH-3T3), human Burkitt's lymphoma cell line (Daudi) and the lymphoid cell line U-266 [33-35]. We also reported that IFN/5-FU induced a marked accumulation of G0-G1 phase by regulating p27kip1 expression in IFN-sensitive human HCC cell line, PLC/PRF/5 [12]. IFN has additional effects on the cell cycle, including S phase prolongation, S phase block and G2/M arrest. Yano *et al*. [36] investigated the anti-proliferative effects of IFN in 13 human HCC cell lines and reported blockade of cell cycle at S-phase in 11 of 13 cell lines. We also examined the effects of IFN or 5-FU on tube formation. Several investigators reported that IFN inhibited endothelial cell tube formation both *in vitro *and *in vivo *[31,32]. Our results confirmed that IFN-α significantly inhibited HUVEC-tube formation *in vitro*, consistent with the previous reports; no additional effect for 5-FU was noted on endothelial cell tube formation.

Does the combination of IFN/5-FU have an indirect anti-angiogenic effect on tumor cells? To answer this question, we examined the concentrations and effects of angiogenic factors released from human HCC cell line, HuH7, in the presence and of absence of IFN and 5-FU and their combination. This approach was based on the fact that angiogenesis is also known to be caused by host and tumor cells reactions. Supernatants from HuH-7 treated with IFN/5-FU significantly inhibited HUVEC growth and induced apoptosis. ELISA assays showed significant reduction of VEGF and Ang-2 and increased Ang-1 in supernatant of IFN/5-FU-treated HUVEC. VEGF and angiopoietins play crucial roles in cancer angiogenesis in various malignancies including HCC. VEGF was initially identified as a vascular permeability factor and is known to evoke proliferation and migration of endothelial cells, and to inhibit apoptosis in pathological angiogenesis [37-39]. VEGF and its receptors are upregulated in various cancers [40] and overexpression of VEGF correlates with microvessel density (MVD), invasiveness and poor prognosis [41]. Angiopoietins are members of endothelial growth factors and have been identified as secreted ligands for receptor-like tyrosine kinase Tie2 [42-44]. Four members of angiopoietins have been detected in recent studies. Ang-1 induces phosphorylation of Tie2 as an agonist and acts as a survival factor for endothelial cells to promote recruitment of pericytes and smooth muscle cells. Ang-2 can also bind with Tie2 but does not induce its phosphorylation. Ang-2 is a biological antagonist and reduces vascular stability. VEGF and angiopoietins play complementary and coordinated roles in vascular development. In the presence of VEGF, Ang-2 promotes vascular sprouting and angiogenesis [45]. High expression of Ang-2 is detected in highly vascular remodeling organs such as the ovaries and placenta. Several investigators reported that the high expression of Ang-2 correlated with MVD or clinicopathological factors in several malignancies including HCC [46-50]. We reported previously that the expression of VEGF and Ang-2 protein correlated with hypervascularity, differentiation and poor prognosis of HCC [50]. Our recent study also showed that IFN/5-FU significantly inhibited *in vivo *angiogenesis of HCC cells with regulation of the VEGF, Ang-1 and Ang-2 expression [16].

To evaluate whether angiopoietins affected by IFN/5-FU play an important role in growth inhibition and apoptosis of HUVEC, we performed rescue experiments with siRNAs knockdown of Ang-1 or Ang-2. Knockdown of Ang-1 abrogated the anti-proliferative effects of the conditioned medium from IFN/5-FU-treated HUVEC while that of Ang-2 resulted in partial rescue. These results suggest that IFN and 5-FU when used in combination regulate the expression of angiopoietins and that these proteins contribute, at least in part, to the anti-angiogenic effects of IFN/5-FU. Further studies are needed to define the exact regulatory mechanisms of angiopoietins by IFN-α and 5-FU and the transcriptional regulation of angiopoietins. IFN-α exerts most of its biological activity by altering the level of gene expression in target cells [51]. Tumor-derived VEGF up-regulates the expression of Ang-2 in host stromal endothelial cells [52]. Battle et al. [53] reported that signal transducer and activator of transcription (STAT) 1, which is one of the signal transducers of IFN, was a negative regulator of angiogenesis and that IFN inhibited Ang-2 expression induced by VEGF. Dickson *et al*. [54] reported recently that IFN directly up-regulated the expression of Ang-1 on tumor cells *in vitro*. It influenced IFN-mediated remodeling of intra-tumoral vasculature and improved drug delivery *in vivo*. These results suggest that regulation of angiopoietins by IFN causes vascular stabilization, reduces vessel permeability and enhances the anti-tumor effects of 5-FU by improvement of drug delivery to tumors.

## Conclusion

We confirmed that the combination of IFN-α and 5-FU had direct anti-proliferative effects on HUVEC and that their synergistic effects were mediated through delays of cell cycle in HUVEC. IFN-α and 5-FU also regulated the expression of VEGF, Ang1 and Ang2 secreted by tumor cells. These actions seem to explain, at least in part, the *in vitro *anti-angiogenic effects of IFN/5-FU, suggesting that they could also contribute to the synergistic anti-tumor effects of these compounds on HCC through remodeling of tumor vasculature and modulating drug delivery.

## List of abbreviations used

5-FU: 5-fluorouracil; Ang: angiopoietin; b-FGF: basic fibroblast growth factor; ELISA: enzyme-linked immunosorbent assay; FdUMP: fluorodeoxyuridine monophosphate; HCC: hepatocellular carcinoma; HUVEC: human umbilical vein endothelial cell; IFN: interferon; IL-8: interleukin-8; MMP: matrix metalloprotease; MVD: microvessel density; PVTT: portal vein tumor thrombus; TUNEL: terminal deoxynucleotidyl transferase-mediated dUTP nick end-labeling; TRAIL: tumor necrosis factor-related apoptosis inducing ligand; VEGF: vascular endothelial growth factor.

## Competing interests

The authors declare that they have no competing interests.

## Authors' contributions

HW, TN and MM were responsible for the molecular genetic studies and performed in vitro experiments. HN, HY, YD and MM contributed to the design of the study, performed the statistical analysis and helped to draft the manuscript. SK, SM, HE, YT, MT and KU contributed to the design of the study and interpretation of the results. All authors read and approved the final manuscript.

## Pre-publication history

The pre-publication history for this paper can be accessed here:

http://www.biomedcentral.com/1471-2407/9/361/prepub
